# Vestibular cognition assessment system: Tablet-based computerized visuospatial abilities test battery

**DOI:** 10.3389/fpsyg.2023.1095777

**Published:** 2023-02-22

**Authors:** Yan Huang, Xuehao Zhang, Jia Tang, Yuqi Xia, Xiaotong Yang, Yanmei Zhang, Chaogang Wei, Ruiqi Ruan, Hang Ying, Yuhe Liu

**Affiliations:** ^1^School of Medical Technology and Information Engineering, Zhejiang Chinese Medical University, Hangzhou, China; ^2^School of Management, Beijing University of Chinese Medicine, Beijing, China; ^3^Department of Otolaryngology, Head, and Neck Surgery, Peking University First Hospital, Beijing, China; ^4^Department of Otolaryngology, Head, and Neck Surgery, Beijing Friendship Hospital, Capital Medical University, Beijing, China

**Keywords:** vestibular dysfunction, visuospatial cognition, cognition, tablet-based test, computerized system

## Abstract

**Introduction:**

The vestibular system is anatomically connected to extensive regions of the cerebral cortex, hippocampus, and amygdala. However, studies focusing on the impact of vestibular impairment on visuospatial cognition ability are limited. This study aimed to develop a mobile tablet-based vestibular cognitive assessment system (VCAS), enhance the dynamic and three-dimensional (3D) nature of the test conditions, and comprehensively evaluate the visuospatial cognitive ability of patients with vestibular dysfunction.

**Materials and methods:**

First, the VCAS assessment dimensions (spatial memory, spatial navigation, and mental rotation) and test content (weeding, maze, card rotation, and 3D driving tests) were determined based on expert interviews. Second, VCAS was developed based on Unity3D, using the C# language and ILruntime hot update framework development technology, combined with the A* algorithm, prime tree algorithm, and dynamic route rendering. Further, the online test was built using relevant game business logic. Finally, healthy controls (HC) and 78 patients with vertigo (VP) were recruited for the VCAS test. The validity of VCAS was verified using the test results of random controls.

**Results:**

In the weeding test, the HC group had a significantly longer span and faster velocity backward than did the VP group. In the 12 × 12 maze, statistically significant differences in step and time were observed between the two groups, with VP taking longer time and more steps. In the mental rotation task, no significant difference was observed between the two groups. Similarly, no significant difference was found in the performance of the two groups on maps 2, 3, and 4 in the 3D driving task.

**Discussion:**

Thus, impaired visuospatial cognition in patients with vestibular dysfunction is primarily related to spatial memory and navigation. VCAS is a clinically applicable visuospatial cognitive ability test for VP.

## 1. Introduction

The vestibular system consists of three semicircular canals and two otolithic organs (the utricle and balloon), all of which contribute to movement stability. Specifically, the vestibulo-ocular reflex maintains clear vision, while the vestibulo-spinal reflex maintains body stability and postural balance during exercise. The vestibular system is anatomically connected to vast regions of the cerebral cortex, hippocampus, and amygdala. The vestibulo-thalamo-cortical pathway transmits spatial information from the vestibular system through the parietal and entorhinal cortices to the hippocampus (Shinder and Taube, 2010; Hitier et al., 2014). Thus, reduced vestibular input could impair these cognitive and affective circuits. Studies have demonstrated that patients with vestibular diseases exhibit cognitive deficits, such as object recognition and memory. Several researchers have conducted cross-sectional studies of the National Health Interview Survey in 2012 and found a corresponding association between vertigo, cognition, and mental illness (Bigelow et al., 2020). In contrast to healthy controls (HC), people with vertigo were three times more likely to have difficulty with mood, concentration, or behavior. Furthermore, patients with vertigo (VP) often have comorbid disorders, such as mental disorders, fatigue, and sleep disorders. However, studies have discovered that short-term memory impairment in VP can occur independently of these disorders (Smith et al., 2019). Despite the various dimensions of cognitive impairment in patients with vestibular dysfunction, research has primarily focused on visuospatial ability and memory, particularly spatial memory, and navigation ability aspects (Bigelow and Agrawal, 2015; Bigelow et al., 2015).

This study used the most available clinical test questionnaires, namely, the dizziness handicap inventory, the vertigo symptom scale, and the vestibular activities and participation. The questionnaires primarily assess the functional, emotional, and physical impacts of vertigo on patients’ daily life, which rarely involve the cognitive dysfunction caused by vertigo (Lacroix et al., 2016). Therefore, cognitive evaluation tools in neuroscience are predominately used to evaluate cognitive function in patients with vestibular dysfunction. Some multi-dimensional testing tools focus on language and memory ability, such as the Montreal cognitive assessment and repeatable battery for the assessment of neuropsychological status. However, visuospatial assessment is considered less in some of these tools. Single-domain tools for assessing visuospatial ability, including the Benton visual retention test (BVRT) and clock drawing test, are predominately limited to assessing one dimension of spatial cognition, such as spatial memory or structural ability. However, most available visuospatial testing tools operate in an offline mode, which makes it impossible to accurately measure reaction time. In these tools, the stimulus presentation is relatively simple, insufficiently dynamic, and unable to control irrelevant variables. Therefore, these traditional scales may have insufficient sensitivity in patients with vestibular dysfunction (Dobbels et al., 2019).

In contrast to other cognitive dimensions tests, visuospatial tests require a greater transmission of sensory information. Furthermore, using three-dimensional (3D) simulations rather than two-dimensional (2D) simulations in perceptual-cognitive tests is more beneficial (Put et al., 2014). Computerized tests can address the lack of sensory stimulation observed in the traditional visuospatial scales, present test scenes in 3D that are more dynamic, and better capture the visuospatial cognitive ability of the participants. Thus, a more realistic experimental scene can be simulated using computer 3D presentation, enriching sensory stimulation (García-Betances et al., 2015). In addition, participants can move their position in a virtual reality environment. This can enhance the simulation of spatial navigation movement, yielding a stronger interactive experience. Navigation tests in real environments are more effective than in virtual reality environments; however, experimental conditions in real environments are variable and uncontrollable. Moreover, offline, real-world environments require a large test site, the management of which is often complicated and time-consuming. Therefore, computerized cognitive tests can mitigate the traditional visuospatial scale’s lack of dynamic and stereoscopic aspects and avoid the disadvantages of the variability of experimental conditions in real environments. Researchers have innovated online visuospatial tests; for example, Morganti (2018) transformed the money roadmap into a virtual reality version, while Claessen et al. (2015) adapted the Corsi block tapping task (CBTT) into a computerized version. Researchers have improved the traditional Morris Water Task and developed a virtual Morris Water Task (vMWT) for humans (Gazova et al., 2013; Daugherty et al., 2015). However, studies have focused on visuospatial cognition problems caused by aging, with less emphasis on the impact of vestibular impairment on visuospatial cognition ability.

According to previous literature, visuospatial domain impairment of patients with vestibular dysfunction is concentrated primarily in the three dimensions of spatial memory, spatial navigation, and mental rotation (Bigelow and Agrawal, 2015; Smith, 2017). Spatial memory denotes the ability to use visual external information and non-visual personal information to store and organize data regarding the surrounding environment (including the relative position, size, and distance of objects), which comprises the basic condition necessary to complete spatial navigation (Iachini et al., 2009). Spatial navigation is a fundamental animal and human behavior that involves planning routes and executing movements toward environmental goals. Many components of successful navigation rely on perception, memory, and executive functions to build spatial representations in the brain, integrate spatial information, and select appropriate navigation strategies. Spatial navigation predominately includes two navigation strategies: self-centered strategy and object-centered strategy (Iachini et al., 2021). Finally, mental rotation refers to an imaginative process in which people use representations to mentally rotate objects in two or three dimensions (Searle and Hamm, 2017). In this study, the conventional visuospatial tests used to evaluate these three dimensions in patients with vestibular dysfunction in previous clinical studies were summarized, screened, improved, and placed online. Finally, a test system was developed for evaluating the three sub-dimensions of visuospatial cognition in patients with vestibular dysfunction.

The present study developed an effective and convenient vestibular cognition assessment system (VCAS). Through this complete, combined testing system, the performance of VP can be efficiently and accurately evaluated simultaneously for the three spatial cognitive sub-dimensions of spatial memory, spatial navigation, and mental rotation. Furthermore, the system can comprehensively assess the visuospatial cognition of patients with vestibular dysfunction. Consequently, VCAS enhances dynamic and rich sensory stimulation and improves the human-computer interaction experience for the participants. Moreover, the test is presented on a mobile tablet terminal, increasing the convenience for the clinician. Particularly, accurate data recording and digital storage of test results facilitate the maintenance and management of patient data. The test results of patients with vestibular dysfunction can be accumulated to perform data mining and big data analysis to assist in smart medical care development.

## 2. Materials and methods

### 2.1. Visuospatial tools summary

The cognitive assessments of patients with vestibular dysfunction published in PubMed, Web of Science, and other literary resources over the past 15 years were reviewed. Consequently, this study discovered that visuospatial dysfunction might comprise the main cognitive impairment in patients with vestibular dysfunction. Table 1 summarizes the use of visuospatial ability testing tools in previous clinical studies that investigated patients with vestibular dysfunction. These tools primarily assess three dimensions of visuospatial ability in patients with vestibular dysfunction: spatial memory, spatial navigation, and mental rotation.

**TABLE 1:** Application of visuospatial tools in patients with vestibular dysfunction in previous clinical studies.

Dimension	Research	Tool
Spatial memory	Bigelow et al. (2015); Popp et al. (2017); Guidetti et al. (2020); Pineault et al. (2020)	Corsi block tapping task, Benton visual retention test
Spatial navigation	Wei et al. (2018); Pineault et al. (2020); Lacroix et al. (2021)	Money road map test, maze task
Mental rotation	Grabherr et al. (2011); Bigelow et al. (2015); Deroualle et al. (2019)	Card rotation test, mental transformation tasks and control task, third-person perspective taking
Spatial memory and navigation	Brandt et al. (2005); Kremmyda et al. (2016)	virtual Morris water task

Despite the widespread use of cognitive tools for visuospatial testing in patients with vestibular dysfunction, these tools have the following limitations:

(1) Most of the assessments are paper and pencil tests. This cannot accurately control the experimental conditions and is inconvenient for managing statistical test data.
(2) Traditional visuospatial scales cannot sufficiently stimulate the senses. Furthermore, the test conditions are predominately composed of static graphics, lack 3D and dynamic sense, and cannot accurately reflect visuospatial cognition.
(3) Some online tests have been improved; however, most are limited to computer presentations, an inconvenient medium for bedside evaluation.
(4) Many tests solely assess one dimension of visuospatial cognition, while few multi-dimensional combined test systems comprehensively evaluate visuospatial ability.

### 2.2. Semi-structured interview

This study invited six experts in vertigo and cognition to discuss the test tools presented in Table 1. Two and four of the six experts were from Peking University First Hospital and Beijing Friendship Hospital, respectively. Based on their in-depth experience in vertigo diagnosis and treatment and spatial cognition assessment, a semi-structured interview was performed with the experts to screen the test methods and indicators with high sensitivity and develop the subsequent test system. Table 2 presents the interview questions.

**TABLE 2:** Interview questions.

Interview outlines
1. Which dimensions do you think should be assessed, and which tests should be included?
2. What existing test or experimental paradigms do you think can be online or improve innovation?
3. In these tests, which indices do you think are more important?
4. In addition to the common indicators, which indicators do you think should be included?

### 2.3. VCAS design

**FIGURE 1:**
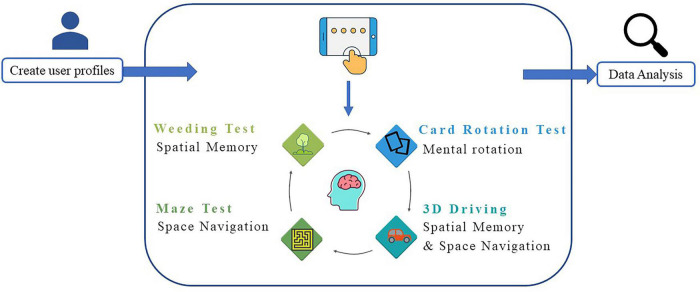
Vestibular cognitive assessment system (VCAS) frame. The system includes three main sections: Information input, test, and result query.

This study designed the VCAS to assess the visuospatial cognitive abilities of VP to assist clinical diagnosis, improve the efficiency of clinicians, and provide guidance for subsequent treatment, care, and rehabilitation. The assessment method, test dimensions of the test system, and the indicators to be collected for each test were determined according to feedback from the interviews (Table 3). The development of the system was user-centered. After several comparative experiments, several visuospatial tests were administered online or modified, resulting in a combined test system suitable for clinical use. Consequently, the VCAS framework was designed in this study (Figure 1). The flat panel has the characteristics of a large visual screen area and high operability. This design used a flat panel to enhance the test experience of the participants. In addition, VCAS identifies relevant research information upon touching the screen and triggers the corresponding event. The touch screen is easier to operate and manage than a keyboard and mouse. Furthermore, it enhances the participants’ sense of mastery and is highly adaptable. Furthermore, the hot update framework development VCAS is an Android-based system built with the 2019 version of Unity3D,^1^ using the C# language and ILRuntime. ILRuntime is a framework used by application software developers that permits hot updates. The C# language version used was 4.x. The system includes three main sections: the information input, test, and result query.

**TABLE 3:** Description of test items and indicator indexes.

Test	Dimension	Index
Weeding test	Spatial memory	Span forward, span backward, velocity forward, velocity backward
Maze test	Spatial navigation	Time, step
Card rotation test	Mental rotation	Score, time
3D driving	Spatial memory and navigation	Response time, errors

3D, three-dimensional.

### 2.4. Information input section

**FIGURE 2:**
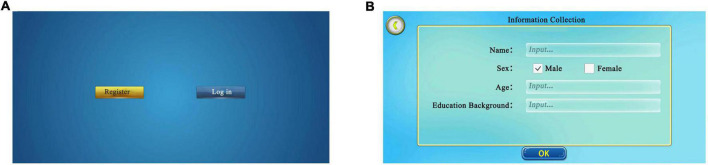
Initial login interface. **(A)** Login interface. New participants select “register”, former participants select “log in” and find their page. **(B)** Information collection. New participants log in and fill in basic information, including name, sex, and age. Adapted from https://unity.com/.

This section contained the participants’ basic information, including demographic information such as name, age, and sex. The confirmation button was clicked to commence the formal test after creating the participant’s profile. Participants with pre-existing profiles could discover their corresponding accounts through the existing database for the formal test (Figure 2).

### 2.5. Test section

#### 2.5.1. Experimental design of the weeding test

**FIGURE 3:**
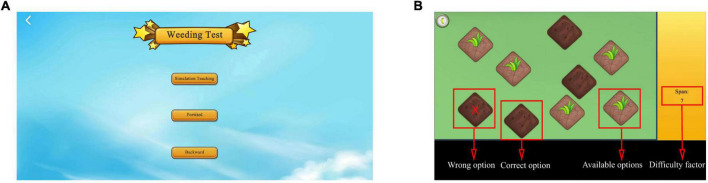
Weeding test. **(A)** Login to the weeding test interface. The weeding test includes forward and backward, and simulation exercises are required before the test. **(B)** Test interface. The screen in the weeding test. Weeds will disappear when selected correctly; when selected incorrectly, the weed will exhibit “×”. Adapted from https://unity.com/.

The weeding test is used to assess spatial memory and is inspired by the CBTT, which determines memory breadth. Specifically, the weeding test is divided into two main sessions, a forward and a backward session. The test uses simulation teaching to help beginners better understand the rules and methods of the test. The American psychologist, George A. Miller, proposed that the maximum capacity of short-term memory lies between 5 and 9 items (Manoochehri, 2021). Based on this hypothesis, the longest span of the weeding game was set to nine in this study. Specifically, a background image of nine sections of grass in the form of squares with weeds growing on them appeared on the screen. The system automatically demonstrated the square jumping, with the jumping interval set to 1 s. After the demonstration, the participants were instructed to reproduce the sequence in the same or reverse order. When participants clicked correctly, the weed on the square automatically disappeared. However, when the participants clicked incorrectly, the weed exhibited an “×” (Figure 3).

The system randomly generated squares to jump, with the number of squares starting at two. Subsequently, the weeding sequence to be memorized gradually increased as the difficulty of the test increased. The sequence length increased progressively; namely, each sequence had two levels, and the game automatically proceeded to the next level when one of the two levels was passed. The game automatically stopped when two sequences of the same level failed or the maximum click limit sequence set by the game was reached. The system automatically registered the dependent variables of the longest series (span) recalled, the total number of blocks clicked, and the total time for the correct item in the forward and backward directions. The weeding test collected two metrics, namely, the longest correct series (span) and the clicking speed (total blocks/total time). Specifically, the longer and faster the longest correct series (span), the better the spatial memory.

#### 2.5.2. Experimental design of the maze test

The maze test was used to evaluate spatial navigation and was inspired by the money road map test and maze task. The maze game used the most basic heuristic global path search A* algorithm for the shortest path solution. The A* algorithm is the most efficient direct search method for shortest-path solving in static routing (Ou et al., 2022). The system randomly generated the corresponding maze map using a prime tree algorithm and dynamic route rendering to avoid learning effects. The principles of the A* algorithm are as follows:

This paradigm consists of per-node priority calculations, for example:


(1) f⁢(n)=g⁢(n)+h⁢(n)

where *n* denotes the node and *f* (*n*) represents the integrated priority of the node *n*. In the process of implementing the algorithm, a smaller value of *f* (*n*) node indicates its higher integrated priority. During the algorithm’s operation, the node with the smallest *f* (*n*) value is preferred. Specifically, *g* (*n*) represents the distance of node *n* from the initial point, and *h* (*n*) represents the distance of node *n* from the target point, where the heuristic function *h* (*n*) is computed using the Manhattan distance.


(2) $$c=\left|x_{1}-x_{2}\right|+\left|y_{1}-y_{2}\right|$$

(3) h⁢(n)=D*c

where *x* and *y* denote the corresponding horizontal and vertical coordinate values of the two nodes, respectively, and *D* denotes the movement cost between the two neighboring nodes.

**FIGURE 4:**
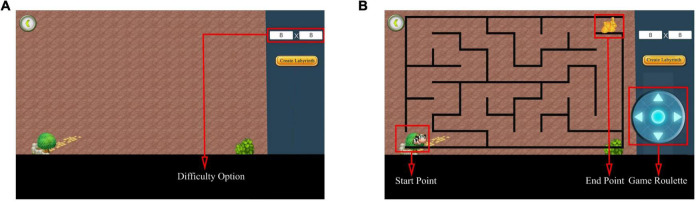
Maze test interface. **(A)** Before creating a maze. Select the difficulty of the maze to be tested by adjusting the difficulty option. **(B)** After creating the maze. The interface after generating the maze using 8 × 8 as an example. Adapted from https://unity.com/.

**FIGURE 5:**
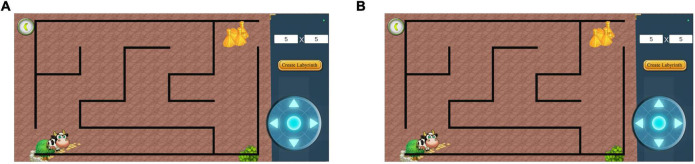
Diagram of different difficulty mazes. **(A)** Difficulty factor 5 × 5 maze. **(B)** Difficulty factor 12 × 12 maze. The higher the difficulty factor, the more complex the maze. Adapted from https://unity.com/.

The researcher created mazes with the starting point of the maze located in the lower left corner, the ending point in the upper right corner, and the game roulette in the lower right corner (Figure 4). The test participants created mazes based on different difficulty factors according to the study’s purpose (Figure 5). After creating the maze, the participant clicked the game roulette and manipulated the cow to move to the end of the maze (Supplementary Figure 1). The total time and number of steps the participant used to complete the maze were automatically registered in the system. Subsequently, the maze test captured the total number of steps and total time as metrics. Specifically, the shorter the total time and the fewer the total number of steps, the better the spatial navigation.

#### 2.5.3. Experimental design of the card rotation test

**FIGURE 6:**
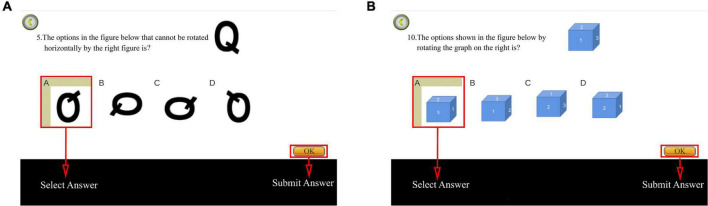
Diagram of card rotation test. **(A)** Two-dimensional (2D) test question. **(B)** Three dimensional (3D) test question. In total, the test comprised eight questions, namely six 2D and two 3D test questions. Adapted from https://unity.com/.

This study used an online card rotation test to assess mental rotation. The test comprised eight questions, divided into six 2D and two 3D test questions (Figure 6). The system provided a reference figure in the upper right corner of the interface, with four figures of the same color and size as the reference figure but with different rotation angles.

The first question was used for the explanation and was not scored. The timing and scoring commenced after the explanation. One point was awarded for each correct answer, with a maximum of eight points. Subsequently, the total score and time were recorded backstage. Consequently, higher scores and shorter time spent indicated improved mental rotation.

#### 2.5.4. Experimental design of the 3D driving test

The 3D driving test was inspired by the vMMT and used to evaluate spatial memory and navigation. The test used the Unity3D engine and accessed the ILruntime hot update framework development to build virtual 3D maps and physical car models. An artificial intelligence pathfinding system was used to realize the car model autopilot function.

**FIGURE 7:**
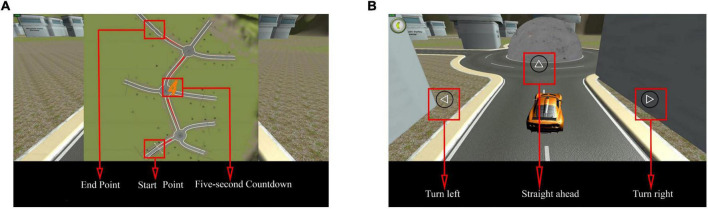
Three dimensional (3D) driving diagram. **(A)** Map style. The map includes the start and end points and is presented in 5 s. **(B)** Road turntable. At the round turntable, participants must choose a direction, including straight ahead, turning left, or turning right. Adapted from https://unity.com/.

**FIGURE 8:**
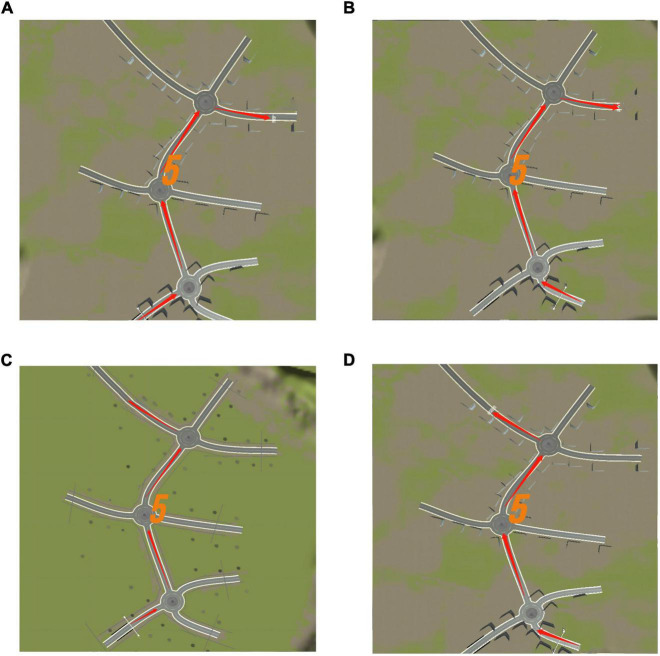
Three dimensional (3D) driving memory map diagram. **(A)** Map 1. **(B)** Map 2. **(C)** Map 3. **(D)** Map 4. Each map has a different starting and ending point. Adapted from https://unity.com/.

The system displayed a map connected to a turntable with three directions at each intersection before the test commenced. The participants were required to memorize the map’s contents within 5 s. After the map disappeared, the participant selected a direction from each intersection based on the memorized content (Figure 7). Each intersection had only one correct direction, and the car could only be driven after the participant had chosen it. The test was designed to ensure that the participant did not need to steer the car to avoid experimental errors caused by the inflexible fingers of some older adults. Particularly, the 3D driving had four maps of the same difficulty level, each with different starting and ending positions (Figure 8). The system recorded the number of selection errors and the response time required to complete the test. Consequently, fewer selection errors and shorter response time indicated better spatial memory and navigation.

### 2.6. Result query section

**FIGURE 9:**
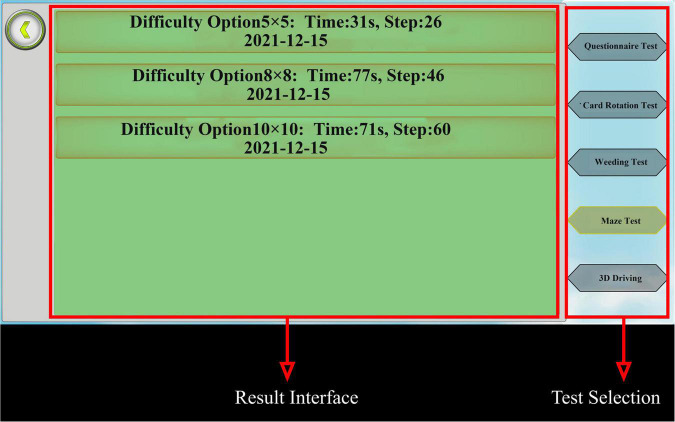
Result display module. Taking the 8 × 8 maze test results as an example, the results include the number of steps and time for different difficulties of the maze test. Adapted from https://unity.com/.

Figure 9 shows the module display with indicators for each test and controls that permit the selection of different tests on the right to view the test results. The system data were stored in the Tencent Cloud storage bucket.

### 2.7. System verification

#### 2.7.1. Participants

In this study, VP were diagnosed with vestibular dysfunction-related diseases at the Department of Otolaryngology and Head and Neck Surgery, Peking University First Hospital, between December 2021 and June 2022. Each patient had a history of dizziness or vertigo, and at least one routine vestibular function test with abnormal results, including:

(1) The cervical vestibular evoked myogenic potential test failed to elicit obvious P1 and N1 waves at 100 dB nHL intensity in monaural or binaural tests (Vanspauwen et al., 2011);
(2) Video-head impulse test with compensatory saccades or abnormal gain (Macdougall et al., 2013);
(3) Unilateral weakness value of > 25 in caloric tests (Shepard and Jacobson, 2016);
(4) Positioning nystagmus evoked in dynamic position test.

The VP group mainly included common vestibular disorders such as otoliths, Meniere’s disease, sudden deafness with vertigo, and vestibular neuritis. The HC were recruited *via* adverts and had no history of dizziness, vertigo, or hearing impairment. For the VP and HC groups, the following exclusion criteria were applied: (1) age < 18 years; (2) inability to understand and cooperate with tests; (3) history of anxiety or depression; (4) related dementia diseases (such as Alzheimer’s disease (AD) and vascular dementia); (5) noticeable visual impairments; (6) motor dysfunction disorder (especially of the upper limbs); (7) central nervous system diseases such as cerebral infarction or neurological disease. We only included adults with concomitant vestibular dysfunction because vestibular disorders are more predominant in adults and are relatively rare in children and adolescents. Moreover, VCAS requires a certain level of cooperation and understanding; thus, we excluded individuals who could not cooperate.

All procedures in this study were approved by the hospital Ethics Committee, and informed consent was obtained from all participants. The test duration for this study was approximately 40 min. Specifically, the weeding, maze, card rotation, and 3D driving tests took approximately 10, 10, 8, and 12 min, respectively. Appropriate breaks were provided, if necessary, during the testing period. This study was conducted in the hospital’s outpatient department. However, some of the tests were not completed because of schedule conflicts for some participants. In the maze test, a maze with a 5 × 5 difficulty was used to familiarize the participants with the test rules. Subsequently, tests of three difficulty levels were conducted, namely 8 × 8, 10 × 10, and 12 × 12. In the 3D driving test, the participants were informed about the test rules using map 1. The tests of maps 2, 3, and 4 were conducted after understanding the rules. The study was conducted on a Lenovo TB-J606F tablet with a resolution of 2,000 × 1,200 and a screen size of 11 inches.

#### 2.7.2. Statistical analyses

SPSS 25.0 was used for the statistical analysis of the data. Quantitative variables were distributed normally using mean (SD) and non-normally using M (P_25_, P_75_). Categorical variables were expressed as frequencies and percentages, n (%). Normality was tested using the Shapiro–Wilk test, and quantitative variables with normal distributions were tested using two independent-sample *t*-tests and those with non-normal distribution using the Mann–Whitney *U* test. For the demographic data, *t*-tests were used to assess age and years of education, and a chi-square test was used to assess sex. Maze and card rotation test data with normal distribution were assessed using *t*-tests, whereas the weeding and 3D driving test data with non-normal distribution were assessed using Mann–Whitney *U* test. Statistical significance was set at *P* < 0.05. GraphPad Prism 9.0 was used to display the overall distribution of the data between the two groups.

## 3. Results

### 3.1. Participants’ characteristics

This study investigated 154 participants: 75 HC (21 males and 54 females) and 79 VP (25 males and 54 females). There were no statistically significant differences between the groups in terms of age (*P* = 0.079), sex (*P* = 0.621), or education (*P* = 0.398; Table 4).

**TABLE 4:** Demographic characteristics of the study population.

	Patients with vertigo (*n* = 79)	Healthy controls (*n* = 75)	
Age: mean (SD)	55.01 (12.05)	51.63 (11.56)	*P* = 0.079
Sex (n, %)			*P* = 0.621
Male	25 (32)	21 (28)	
Female	54 (68)	54 (72)	
Educational level: mean (SD)	12.77 (2.56)	12.38 (2.99)	*P* = 0.398

### 3.2. Comparison of the results of the HC and VP groups

#### 3.2.1. Comparison of weeding test results

**FIGURE 10:**
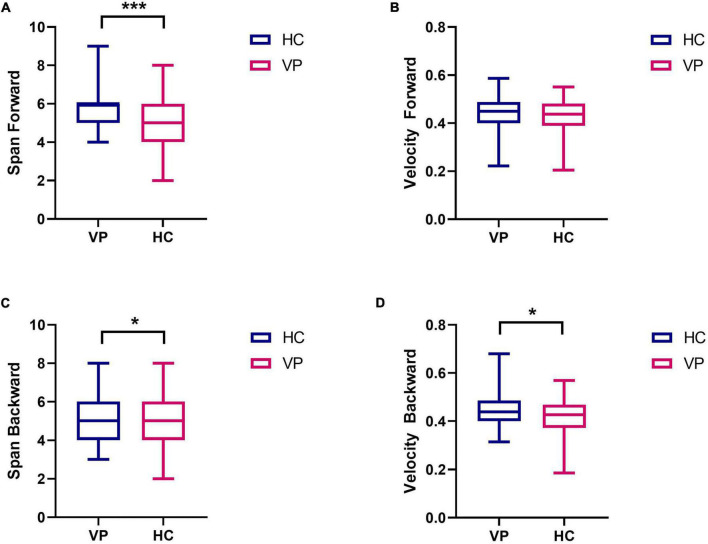
Comparison of weeding test indexes between VP and HC. **(A)** Span forward. **(B)** Velocity forward. **(C)** Span backward. **(D)** Velocity backward. The upper and lower lines represent the 95th and 5th percentiles, respectively, and the horizontal line within the box is the median. VP, patients with vertigo; HC, healthy controls. ***P < 0.01, *P < 0.05.

All the participants completed the weeding test, and the Mann–Whitney *U* test was used to compare the results between the two groups. As shown in Figure 10 and Supplementary Table 1, the median span forward was 5 (4.00, 6.00) for the VP group and 6 (5.00, 6.00) for the HC group; the difference was significant (*z* = –3.85, *P* < 0.001). Similarly, a significant difference was observed in the span backward between the groups (*z* = –1.97, *P* < 0.05). However, the median velocity forward was 0.44 (0.39, 0.48) for the VP group and 0.44 (0.40, 0.49) for the HC group; the difference was insignificant (*z* = –0.86, *P* = 0.392). Finally, the VP group had a lower negative weeding rate than the HC group (*P* < 0.05).

#### 3.2.2. Comparison of maze test results

**FIGURE 11:**
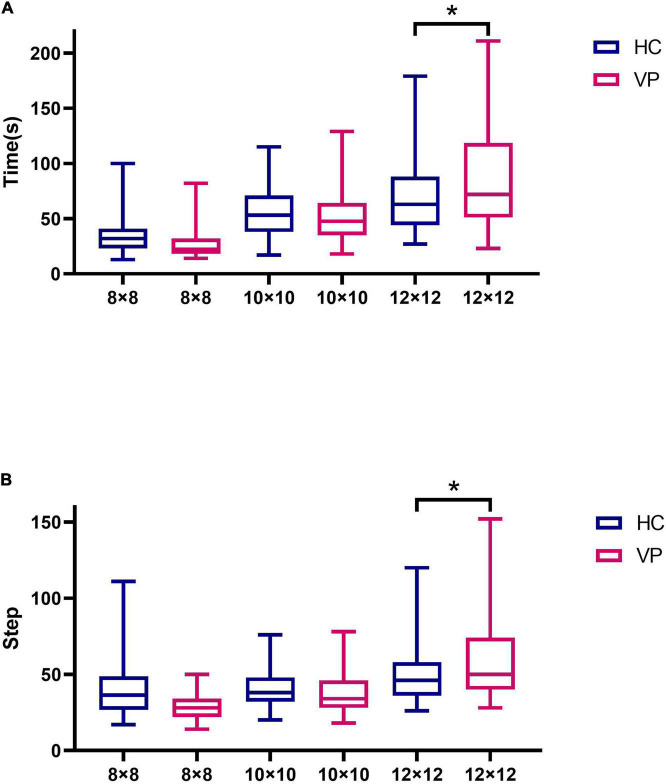
Comparison of maze test indexes between VP and HC. **(A)** Time spent in maze tests of 8 × 8, 10 × 10, and 12 × 12 difficulty for VP and HC, respectively. **(B)** The number of steps in maze tests of 8 × 8, 10 × 10, and 12 × 12 difficulty for VP and HC, respectively. The upper and lower lines represent the 95th and 5th percentiles, respectively, and the horizontal line within the box is the median. VP, patients with vertigo; HC, healthy controls. *P < 0.05.

In total, 67 HC and 66 VP completed the 8 × 8, 10 × 10, and 12 × 12 maze tests in this study. Ten individuals did not complete the weeding test because of schedule conflicts. For the 8 × 8 maze, the mean times (s) for the VP and HC groups were 40.48 (18.26) and 34.75 (15.97), respectively, with no significant difference between the groups (*t* = 1.93, *P* = 0.056). Further, for the 8 × 8 maze, the mean steps of the VP and HC groups were 28.39 (8.63) and 27.10 (11.53), respectively; the difference was insignificant (*t* = 0.73, *P* = 0.467). For the 10 × 10 maze, the mean times (s) and steps of the VP and HC groups showed no significant difference (*P* > 0.05). For the 12 × 12 maze, the mean times (s) of the VP and HC groups were 85.76 (48.35) and 69.57 (31.01), respectively, and the mean steps were 58.06 (25.20) for the VP group and 49.52 (16.45) for the HC group; all showed significant differences (*P* all < 0.05). Figure 11 and Supplementary Table 1 show the maze test results for the two groups.

#### 3.2.3. Comparison of the card rotation test results

**FIGURE 12:**
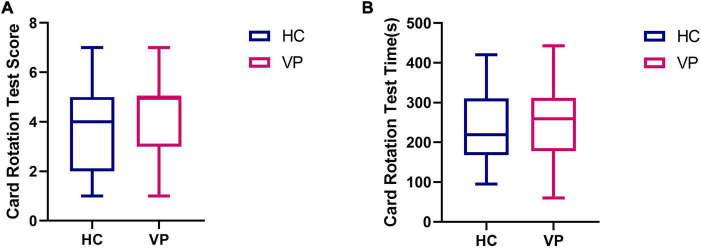
Comparison of card rotation test indexes between VP and HC. **(A)** Score. **(B)** Time (s). The upper and lower lines represent the 95th and 5th percentiles, respectively, and the horizontal line within the box is the median. VP, patients with vertigo; HC, healthy controls.

Due to the pre-experimental replacement of some question items, only 41 HC and 38 VP completed the card rotation test in this study. These results are shown in Figure 12 and Supplementary Table 1. The mean scores were 4.31 (1.65) for the VP group and 3.84 (1.65) for the HC group; the difference was insignificant (*t* = 1.28, *P* = 0.205). The mean times (s) were 252.21 (99.62) for the VP group and 234.88 (98.70) for the HC group; the difference was insignificant (*t* = 0.655, *P* = 0.515).

#### 3.2.4. Comparison of the 3D driving test results

**FIGURE 13:**
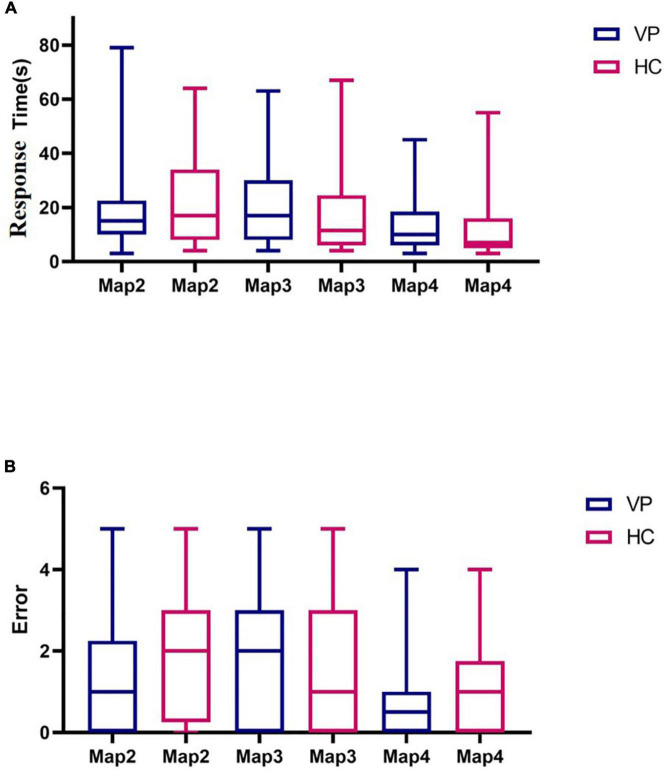
Comparison of three dimensional (3D) driving test indexes between VP and HC. **(A)** Response time spent in Maps 2, 3, and 4 for VP and HC, respectively. **(B)** The number of errors in Maps 2, 3, and 4 of VP and HC, respectively. The upper and lower lines represent the 95th and 5th percentiles, respectively, and the horizontal line within the box is the median. VP, patients with vertigo; HC, healthy controls.

Maps 2, 3, and 4 were completed by 42 HC and 40 VP, 40 HC, and 39 VP, and 42 HC and 40 VP participants, respectively. The reason for this was that we initially only had map 1, and we subsequently added maps 2, 3, and 4. We compared the response time and the number of errors between the two groups, and because these data did not conform to a normal distribution, we used the Mann–Whitney *U* test. In map 2, the median response time (s) of the VP and HC groups were 17.00 (8.00, 33.25) and 15.00 (10.00, 22.50), respectively, with no significant difference observed (*z* = 0.84, *P* = 0.399). Furthermore, the median errors of the VP and HC groups for map 2 were 1.50 (0.00, 2.25) and 1.40 (1.27), respectively, with no significant difference (*z* = 0.90, *P* = 0.371). For map 3, the median response times (s) of the VP and HC groups were 11.50 (6.00, 24.50) and 17.00 (8.00, 30.00), respectively, with no significant difference (*z* = 1.19, *P* = 0.235). The median errors of the VP and HC groups for map 3 were 1.00 (0.00, 3.00) and 2.00 (0.00, 3.00), respectively, with no significant difference (*z* = 1.35, *P* = 0.177). However, we found no difference between the two groups in terms of response time and the number of errors in map 4 (*P* > 0.05) (Figure 13 and Supplementary Table 1).

## 4. Discussion

Animal and clinical studies have indicated that visuospatial abilities are the most relevant in vestibular dysfunction. In a clinical study, Guidetti et al. (2020) used CBTT to evaluate 263 patients with vestibular dysfunction and 430 healthy people. Their study results revealed that the CBTT span was significantly lower in patients than in HC. Furthermore, they discovered that vestibular disease significantly influenced spatial working memory ability. Pineault et al. (2020) used the BVRT to evaluate the spatial memory of participants. The experimental results demonstrated that the BVRT errors were significantly higher in patients with bilateral semicircular canal injury than in the HC group. Moreover, Kremmyda et al. (2016) found that in contrast to the HC, patients with bilateral vestibulopathy (BVP) took longer to reach the exit in the vMWT task and exhibited significant difficulties in spatial memory and navigation. Consistent with these findings, Brandt et al. (2005) used magnetic resonance imaging technology on 10 patients with chronic BVP and discovered that the hippocampus was significantly atrophied. In the vMWT task, patients with BVP exhibited significant heading deviation errors. Researchers in related animal studies also underscored the impact of vestibular impairment on visuospatial cognition. A study found that rats with chronic unilateral vestibular dysfunction had impaired spatial memory during foraging tasks in a dark environment (Zheng et al., 2006). In addition, several studies have demonstrated impaired hippocampal function in rats with bilateral vestibular deafferentation, affecting their spatial learning and memory (Zheng et al., 2007). Similarly, Nguyen et al. (2021) found that unilateral labyrinthectomy impairs spatial memory, navigation, and motor coordination in mice.

The influence of vertigo on cognitive function is an emerging field and represents a novel scientific problem related to cognition, discovered by researchers. For basic research and clinical application, developing and implementing tools to detect the impact of vestibular dysfunction on cognitive function is necessary. VCAS serves as a comprehensive and objective test system to detect the effects of vertigo disorders and vestibular dysfunction on cognitive function. In contrast to previous cognitive function assessments, the VCAS permits comprehensive detection and evaluation of the dimensions underlying visuospatial cognitive ability, such as spatial memory, spatial navigation, and mental rotation. These aspects specifically pertain to the effects of vertigo and vestibular dysfunction on cognitive function. Furthermore, visuospatial cognition research in clinical and related fields is important because it can provide clinical tools for spatial cognitive ability testing with a high clinical diagnostic and early warning value. Lacroix et al. (2021) preliminarily explored spatial cognitive assessment for children with vestibular dysfunction. In contrast, this study investigated adult patients with vestibular dysfunction, emphasizing older adults. In addition, we hope that VCAS will be used in the future to assess visuospatial cognition in patients with other diseases, such as mild cognitive impairment (MCI) or AD. Moreover, studies have shown that visuospatial cognitive testing may be a reliable technique and screening tool for identifying MCI or AD (Lester et al., 2017; Li et al., 2022). Plácido et al. (2021) found that compared with the Mini-Mental State Examination, the visuospatial test had better sensitivity for distinguishing patients with AD from HC, indicating that visuospatial cognitive decline could be independent of general cognitive decline. In addition, Wei et al. (2019) found that the odds of impaired vestibular function were three to four times higher in patients with MCI compared to HC. Further, the degree of vestibular impairment was higher in patients with AD than in controls and patients with MCI. Hence, a relationship might exist between AD, vestibular dysfunction, and cognitive decline (Agrawal et al., 2020). Therefore, the VCAS should be modified and updated accordingly to discover a convenient screening version for AD or MCI. For example, patients with MCI or AD should only perform the 8 × 8 maze test, and the number of questions in the card rotation test should be reduced. In addition, they should be able to perform the weeding test; however, the 3D driving test might be skipped for now to save time. Hence, further exploration and verification are required.

First, the experimental design of the mobile-based VCAS can improve clinicians’ efficiency. Among these designs, the weeding test was inspired by the experimental paradigm of CBTT, which is used to evaluate spatial memory ability. Upon computerizing CBTT, sensory stimulation was primarily provided by flashing square lights (Brunetti et al., 2014; Claessen et al., 2015). In VCAS, the sensory stimulus for the weeding test is provided using “dancing” cubes. As opposed to simple visual light stimulation, jumping cubes provide a stronger sense of spatial dynamics, which can better reflect the role of the vestibular system. The maze test evaluated participants’ spatial navigation abilities and used the A* algorithm combined with the prime tree algorithm and dynamic route rendering to randomly generate different mazes with the same difficulty level, avoiding learning effects and thereby improving test accuracy. The maze task developed by Lacroix et al. (2021) can only be completed using an electronic pen to draw lines. In contrast, the maze test of VCAS allows the simulated villain to move by manipulating the direction arrows. This can enhance the simulation of spatial navigation in real life and interactivity. The card rotation test evaluated the participants’ mental rotation ability using 2D and 3D graphics. The 3D driving test evaluated the spatial memory and navigation ability of the participants and used the Unity3D engine and ILruntime hot update framework development technology to enhance the testing experience for the participants. Compared with traditional paper-and-pencil tests, VCAS digitally stores the data of patients. Capturing, analyzing, and predicting these electronic medical record data is possible by continuously accumulating the data of patients with vestibular dysfunction, permitting more direct and accurate diagnosis and treatment suggestions for clinical practice and rich data support for scientific research (Gamache et al., 2018). We hope that in the future, VCAS will be useful for cognitive screening and the detection of issues in patients with vestibular dysfunction.

Second, the test results indicated that the spatial memory performance of the VP in the backward weeding test was worse than that of the HC. In the forward weeding, no difference in velocity was observed between the groups, whereas the HC group had a shorter span forward than the VP group, indicating that vestibular dysfunction affected spatial memory ability. Popp et al. (2017) also found that patients with BVP performed worse than normal controls in CBTT. Furthermore, the maze test revealed no significant differences in steps or times between the VP and HC for the two difficulties of the 8 × 8 and 10 × 10 mazes. However, the 12 × 12 maze exhibited significant differences in the steps and times between the two groups, with VP requiring more time and steps. Consequently, this suggests that the 12 × 12 maze is more sensitive than the 8 × 8 and 10 × 10 mazes. In the mental rotation test, no significant difference was found between the groups, which could be attributed to the relatively few stimuli, and hence poor sensitivity. Thus, future studies should appropriately increase the number of questions for card rotation and establish a corresponding test question bank. In contrast to spatial memory and navigation, vestibular dysfunction may have a relatively lower impact on mental rotation. This may be because the head orientation and position cells in the hippocampus may receive more vestibular information, whereas the retrosplenial cortex, which is responsible for mental rotation, may be less influenced by vestibular information (Hitier et al., 2014). In the 3D driving test, no significant difference was found in the performance of the two groups on maps 2, 3, and 4, which could be due to the low number of patients with chronic vertigo among the VP. Previous studies have revealed that chronic unilateral vestibulopathy or patients with BVP and persistent postural-perceptual dizziness significantly affect brain structure and function, while cognitive impairment may be more significant (Li et al., 2020; Si et al., 2022). Therefore, more data on patients with chronic vertigo should be collected for future comparative experiments.

This study also demonstrated that patients with vestibular dysfunction could experience changes in cognitive function in addition to their balance and sensorimotor disorders. Thus, the routine balance exercise rehabilitation of patients with vestibular dysfunction should be prioritized. Furthermore, research ought to actively advocate for corresponding cognitive assessment and rehabilitation. Age is a common cause of the cognitive decline. However, no significant difference was found in age between the two groups in this study, in which experimental errors due to age factors were avoided. In addition to age factors, this experiment excluded other diseases affecting cognitive function, such as dementia. Notably, hearing loss could also mediate cognitive decline, increasing the risk of dementia (Kim et al., 2018; Maharani et al., 2018). Thus, this study excluded people with moderate or higher levels of hearing loss while controlling for the influence of hearing factors. Moreover, this study has clinical value and indicates the social significance of evaluating and identifying risk factors for cognitive function in VP.

This research had several limitations: (1) The VP recruited in this study had a relatively short disease course and a mild degree of vertigo. Consequently, these participants could not be distinguished from HC in some tests. (2) The test trials must be made more intensive, such as increasing the response time in the maze test and total number of trials in the weeding test to ensure that each participant answers before the test is terminated. (3) The VP were not classified according to disease. In the future, the performance of people with different vestibular diseases for each visuospatial dimension and the impact of different vestibular organ damage on visuospatial cognition could be explored.

## 5. Conclusion

In conclusion, VCAS can assess the visuospatial abilities of VP in multiple dimensions and at multiple levels. VCAS can provide more 3D and dynamic simulation conditions than traditional visuospatial tests. Vertigo research is a growing discipline and has developed rapidly in recent years. Furthermore, vertigo diseases primarily involve neurology, otolaryngology, head, and neck surgery, neurosurgery, and orthopedics. Despite significant advances in understanding vertigo disease and its related effects, there are many areas to explore and study in the future. The research team that conducted this study is also developing a corresponding visuospatial clinical screening questionnaire, which will be embedded in the test system in the future to permit subjective and objective evaluations. Currently, VCAS is used primarily on mobile phones or tablets, which are convenient to carry. Hopefully, devices can be interconnected in the future so that test results can be accessed across several devices. Moreover, according to clinical needs, a corresponding electronic report is automatically generated, which is convenient for printing and enables timely response to patients.

## Data availability statement

The raw data supporting the conclusions of this article will be made available by the authors, without undue reservation.

## Ethics statement

The studies involving human participants were reviewed and approved by the Ethical Committee of Peking University and the Peking University First Hospital (Approval no. 2021-390).

## Author contributions

YH: experimental design, data collection, data processing, and manuscript writing. JT, XZ, and YX: data processing. XY, YZ, CW, RR, and HY: data collection. YL: experimental design and project implementation management. All authors contributed to the article and approved the submitted manuscript version.

## Footnotes

1
https://docs.unity.cn/cn/current/Manual/index.html
