# Supplementary material for: Vestibular cognition assessment system: Tablet-based computerized visuospatial abilities test battery
Source: Front Psychol. 2023 Feb 22;14:1095777. doi: 10.3389/fpsyg.2023.1095777 (PMC9992172; doi:10.3389/fpsyg.2023.1095777)
Supplement: Supplementary file 1 [file Table_1.docx]

Supplementary Material

**Supplementary Table S1.** Comparison of Vestibular Cognitive Assessment System (VCAS) indexes between vertigo patients and healthy controls.

| **Indexes** | **Patients with vertigo** | | | **Healthy controls** | ***t/z*-value** | ***P*-value** |
| --- | --- | --- | --- | --- | --- | --- |
| **Weeding test indexes: M (P_25_, P_75_)** | |  | |  |  |  |
| Span forward | | 5 (4.00, 6.00) | | 6 (5.00, 6.00) | -3.85 | **<0.001** |
| Velocity forward | | 0.44 (0.39, 0.48) | | 0.44 (0.40, 0.49) | -0.86 | 0.392 |
| Span backward | | 5 (4.00, 6.00) | | 5 (4.00, 6.00) | -1.97 | **0.049** |
| Velocity backward | | 0.43 (0.37, 0.47) | | 0.44 (0.40, 0.49) | -2.11 | **<0.035** |
| **Maze test indexes: mean (SD)** | |  | |  |  |  |
| 8 × 8 Time (s) | | 40.48 (18.26) | | 34.75 (15.97) | 1.93 | 0.056 |
| 8 × 8 Step | | 28.39 (8.63) | | 27.10 (11.53) | 0.73 | 0.467 |
| 10 × 10 Time (s) | | 51.48 (22.41) | | 55.75 (24.36) | -1.05 | 0.296 |
| 10 × 10 Step | | 38.45 (13.15) | | 40.51 (12.18) | -0.93 | 0.352 |
| 12 × 12 Time (s) | | 85.76 (48.35) | | 69.57 (31.01) | 2.30 | **0.024** |
| 12 × 12 Step | | 58.06 (25.20) | | 49.52 (16.45) | 2.31 | **0.023** |
| **Card rotation test indexes: mean (SD)** | |  |  | |  |  |
| Score | | 3.84 (1.65) | 4.31 (1.65) | | -1.28 | 0.205 |
| Time (s) | | 234.88 (98.70) | 252.21 (99.62) | | -0.655 | 0.515 |
| **3D driving test indexes: M (P_25_, P_75_)** | |  |  | |  |  |
| Map 2 Response time (s) | | 17.00 (8.00, 33.25) | 15.00 (10.00, 22.50) | | 0.84 | 0.399 |
| Map 2 Errors | | 1.50 (0.00, 2.25) | 1.00 (0.00, 2.00) | | 0.90 | 0.371 |
| Map 3 Response time (s) | | 11.50 (6.00, 24.50) | 17.00 (8.00, 30.00) | | 1.19 | 0.235 |
| Map 3 Errors | | 1.00 (0.00, 3.00) | 2.00 (0.00, 3.00) | | 1.35 | 0.177 |
| Map 4 Response time (s) | | 7.00 (4.75, 13.00) | 10.00 (6.00, 18.50) | | 0.80 | 0.425 |
| Map 4 Errors | | 0.00 (0.00, 1.00) | 0.00 (0.00, 1.00) | | 0.31 | 0.760 |

3D, three dimensional.

# Supplementary Figure

**
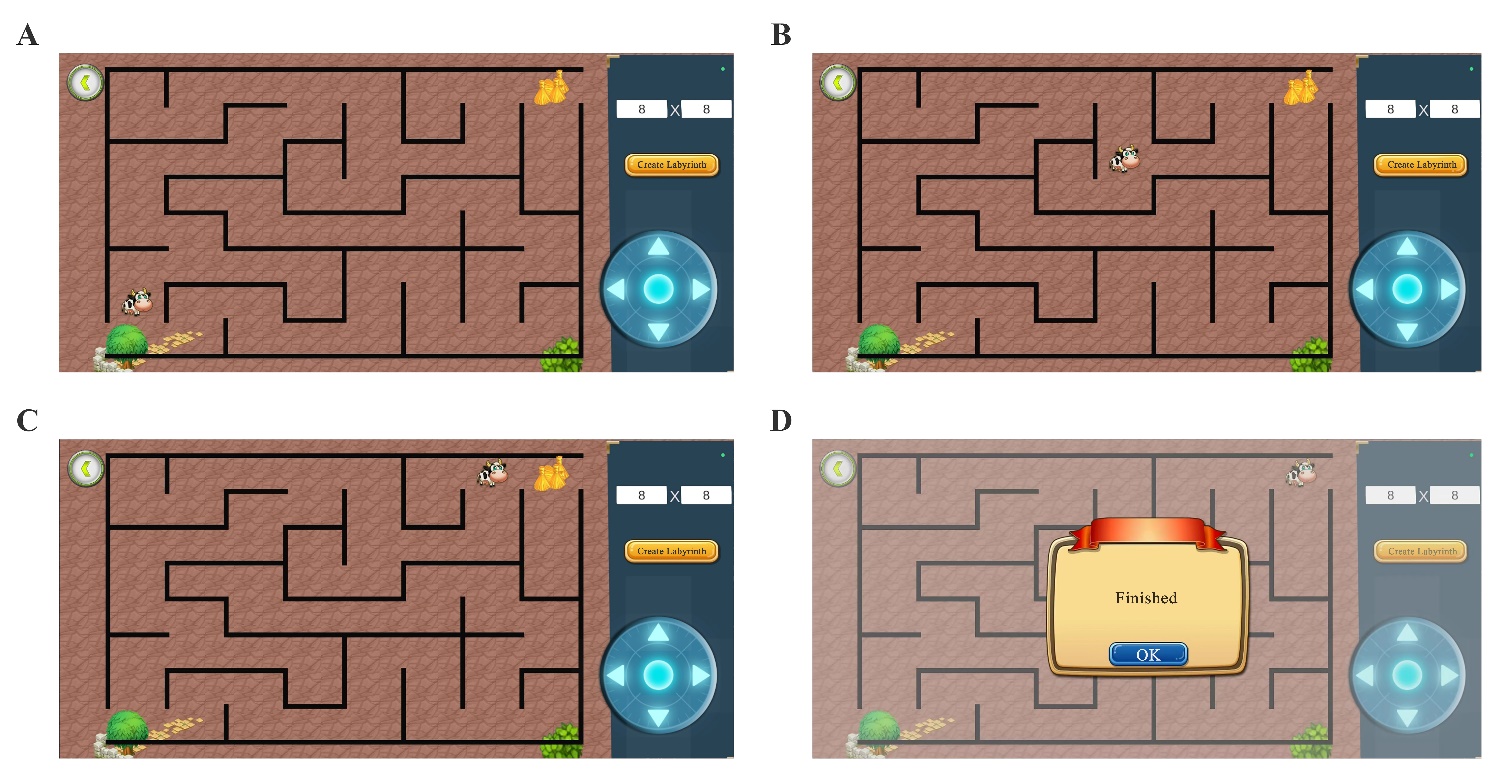
**

**Supplementary Figure S1. Labyrinth test motion track demonstration. (A)** Start the game. **(B)** Halfway through the game. **(C)** Reach the end of the game. **(D)** Complete the game. Adapted from https://unity.com/.
